# Patient-Reported Wait Times and the Impact of Living with Chronic Pain on their Quality of Life: A Waiting Room Survey in Chronic Pain Clinics in Ontario, Manitoba, and Quebec

**DOI:** 10.1080/24740527.2024.2345612

**Published:** 2024-04-22

**Authors:** Clare Liddy, Lynn Cooper, Geoff Bellingham, Tracy Deyell, Pablo Ingelmo, Isabella Moroz, Patricia Poulin, Alexander Singer, Gabrielle S. Logan, Regina Visca, Amin Zahrai, Norman Buckley

**Affiliations:** a Department of Family Medicine, University of Ottawa, Ottawa, Ontario, Canada; b C.T. Lamont Primary Health Care Research Centre, Bruyère Research Institute, Ottawa, Ontario, Canada; c Ontario eConsult Centre of Excellence, The Ottawa Hospital, Ottawa, Ontario, Canada; d Canadian Injured Workers Alliance, Thunder Bay, Ontario, Canada; e Department of Anesthesia & Perioperative Medicine, Western University, London, Ontario, Canada; f Edwards Family Interdisciplinary Center for Complex Pain, Division of Pediatric Anesthesia, Montreal Children’s Hospital, Montreal, Quebec, Canada; g Department of Anesthesia, McGill University, Montreal, Quebec, Canada; h Department of Anesthesiology, The Ottawa Hospital Pain Clinic, Ottawa, Ontario, Canada; i Clinical Epidemiology, The Ottawa Hospital Research Institute, Ottawa, Ontario, Canada; j Department of Anesthesiology & Pain Medicine, University of Ottawa, Ottawa, Ontario, Canada; k Department of Family Medicine, University of Manitoba, Winnipeg, Manitoba, Canada; l Department of Anesthesiology, Perioperative, and Pain Medicine, Max Rady College of Medicine, University of Manitoba, Winnipeg, Manitoba, Canada; m Department of Family Medicine, Faculty of Medicine, McGill University, Montréal, Quebec, Canada; n RUISSS McGill Centre of Expertise in Chronic Pain, Montréal, Quebec, Canada; o Department of Anesthesia, McMaster University, Hamilton, Ontario, Canada

**Keywords:** Chronic pain, wait times, patient experience, quality of life

## Abstract

**Background:**

Wait times at Canadian multidisciplinary pain clinics have been reported as excessive for nearly 2 decades.

**Aims:**

The aim of this study was to gain insight into the patient experience of waiting for chronic pain specialty care.

**Methods:**

A cross-sectional survey of new patients waiting for an appointment was conducted in six multidisciplinary pain clinics, including one pediatric clinic, in Ontario, Quebec, and Manitoba between February 2020 and October 2022. Participants were asked about the length of time they waited for their appointment since being referred, their quality of life, health care professionals seen while waiting, and an open-ended question, “Is there anything else you’d like to tell us?”

**Results:**

Among the 493 adult and 100 pediatric respondents, 53% of adults and 82% of children reported wait times under 6 months, whereas 22% of adults and 4% of children waited longer than a year. Between 52% and 63% of adults and 29% to 48% of children reported being affected by chronic pain “quite a bit” or “extremely” on measures of quality of life. The most visited health care professionals while waiting for a pain clinic appointment were family doctors/nurse practitioners for adults and physiotherapists for children. Qualitative analysis of open-ended question responses revealed eight themes: system navigation issues, administrative issues, decreased quality of life, distress, self-advocacy, coping strategies, communication, and distrust.

**Conclusions:**

Our findings provide real-time regional snapshots into the impact of long wait times experienced by Canadians living with chronic pain. There is an urgent need to better support patients during the waiting period. Expanding technologies such as electronic consultation hold great promise.

## Résumé

**Contexte**: Les temps d’attente dans les cliniques multidisciplinaires de traitement de la douleur au Canada sont jugés excessifs depuis près de deux décennies.

**Objectifs:** L’objectif de cette étude était de mieux comprendre l’expérience des patients en attente de soins spécialisés pour la douleur chronique.

**Méthodes:** Une enquête transversale sur les nouveaux patients en attente d’un rendez-vous a été menée dans six cliniques multidisciplinaires de traitement de la douleur, dont une clinique pédiatrique, en Ontario, au Québec et au Manitoba, entre février 2020 et octobre 2022. Les participants ont été interrogés sur le temps d’attente pour leur rendez-vous depuis qu’ils avaient été référés, sur leur qualité de vie, sur les professionnels de la santé qu’ils avaient consultés pendant qu’ils attendaient, et sur une question ouverte : « Y a-t-il autre chose que vous aimeriez nous dire? »

**Résultats:** Parmi les 493 adultes et les 100 enfants interrogés, 53 % des adultes et 82 % des enfants ont déclaré des temps d’attente inférieurs à six mois, tandis que 22 % des adultes et 4 % des enfants ont attendu plus d’un an. Entre 52 % et 63 % des adultes et 29 % à 48 % des enfants ont déclaré être affectés par la douleur chronique « assez » ou « extrêmement » sur les mesures de la qualité de vie. Les professionnels de la santé les plus consultés pendant l’attente d’un rendez-vous à la clinique de la douleur étaient les médecins de famille/infirmières praticiennes pour les adultes et les physiothérapeutes pour les enfants. L’analyse qualitative des réponses aux questions ouvertes a révélé huit thèmes : les problèmes de navigation dans le système; les problèmes administratifs; la diminution de la qualité de vie; la détresse; l’autonomie; les stratégies d’adaptation; la communication et la méfiance.

**Conclusions:** Nos résultats offrent des instantanés régionaux en temps réel des répercussions des longs temps d’attente sur les Canadiens souffrant de douleur chronique. Il est urgent de mieux soutenir les patients pendant la période d’attente. Les technologies en expansion, telles que la consultation électronique, sont très prometteuses.

## Introduction

Chronic pain is defined as any pain lasting longer than 3 months.^1,2^ It affects one in five adults and children in Canada^3,4^ and globally.^5^ Patient populations with higher prevalence of chronic pain include women^6−8^ and older adults.^8,9^ Chronic pain also disproportionately impacts other equity seeking groups, such as people with lower socioeconomic position,^10^ people living with mental health and substance use disorders,^11^ Indigenous peoples,^12^ certain ethnic and racialized communities,^13,14^ veterans,^15^ and sexually and gender-diverse persons.^16^ When not effectively managed, chronic pain has detrimental impacts on all aspects of quality of life in every age group and for every type of pain.^17^ The total direct and indirect (related to loss of productivity) costs associated with chronic pain in Canada reached $38.3 to $40.4 billion in 2019.^18^ Individuals living with chronic pain often face long wait times for specialist consultation, diagnostic testing, and treatments, which have negative consequences on their physical and mental health.^19^ These negative consequences include diminished quality of life; reduced productivity; lost wages; worsening of chronic disease and psychiatric disorders such as depression, anxiety, and substance use disorders; as well as increased risk for suicide and suicidal ideation.^1,20,21^ In Canada, the median wait time for the first appointment in a publicly funded multidisciplinary pain treatment facility has remained at around 6 months for the last 18 years but can be as long as 4 to 5 years.^22,23^ Living with chronic pain affects one’s ability to fully participate in daily activities and carries a wide range of impacts on families, communities, and the health care system.^24^ Abundant research evidence also indicates that people living with chronic pain are prone to stigmatizing reactions of others.^25,26^ These in turn have a detrimental impact on the physical and psychological well-being of those who are stigmatized.

This study is part of a large-scale research and implementation project focusing on expanding electronic consultation (eConsult) for chronic pain to improve patient access to chronic pain expertise in three provinces—Ontario, Quebec, and Manitoba, which are early adopters of eConsult service. Our specific objective is to fill the gap around the lack of knowledge on how patients living with chronic pain are affected by the waiting period to access and receive services at the multidisciplinary pain clinics by directly examining their experiences and perspectives regarding current wait times and the impact of waiting with chronic pain on their daily activities and quality of life. This knowledge is important to guide the development and implementation of effective measures to improve patients’ experiences of access to care and services for their chronic pain. The results will directly inform the development of an effective eConsult service in each province and across Canada.

## Materials and Methods

### Setting

Our study was conducted at six academic chronic pain clinics across three provinces in Canada: Ontario (Hamilton, London, and Ottawa), Manitoba (Winnipeg), and Quebec (Montreal).

### Participants

New patients attending or waiting to attend a scheduled appointment or clinic orientation session at any of the six participating chronic pain clinics between February 2020 and October 2022 were eligible to participate in the study. The inclusion criteria were the following: new patients (inclusive of both in-person and virtual visits), over the age of 18, not acutely ill or cognitively impaired, and able to complete the survey in English or French. Montreal Children’s Hospital was the only pediatric clinic, with participants under 18 years of age and for whom the survey was filled out by the guardians of the participants, not the participants themselves. Each of the six participating sites aimed to recruit 100 participants over the study period.

### Survey Instrument

Study participants were given a 20-item survey, available in English and French, which took approximately 10 to 15 min to complete (see Appendix A, Supplementary material 1). The survey was adapted from our previous waiting room survey of patients living with chronic pain^21^ and modified through several interactive meetings and discussions with our patient partners using a co-design approach.^27^ The survey collected basic demographic information (age and gender) but did not collect any identifying personal health information. Questions in the survey pertained to patients’ chronicity and impact of pain symptoms, wait times, burden of current wait times, and health care services accessed while waiting. Participants were invited to also leave additional comments in an open-text field.

### Recruitment Procedure

Due to the COVID-19 pandemic and the numerous accompanying restrictions on in-person visits and process changes in health care settings, including chronic pain clinics,^28^ the number of new patient visits varied by site. Each study site adapted differently to the pandemic and therefore had a unique participant recruitment strategy and timeline based on individual research ethics board requirements, clinic administrative capabilities, and clinic capacity. The study was approved by the Ottawa Health Science Network Research Ethics Board (OHSN-REB, Protocol ID#: 20190385–01H) and the Bruyère Continuing Care Research Ethics Board (Bruyère REB, Protocol #M16-19033) and received individual approvals from the research ethics boards of each participating institution (McGill University Health Center Research Ethics Board #2020-5902, University of Manitoba Health Research Ethics Board #HS23112 [H2019:32], Western University Health Science Research Ethics Board #114585, and Hamilton Integrated Research Ethics Board #7800).

To recruit study participants, new patients attending the chronic pain clinics were approached by a member of either the clinic or research team. Depending on the COVID-19 protocols at the local site, this was done in person on the day of their first appointment, remotely via phone, or through mail. The inclusion criteria were as follows: (1) new patient to the clinic, (2) 18 years of age or older (except in the pediatric clinic), and (3) able to speak/comprehend English or French well enough to provide informed consent and participate. Regardless of whether in-person or remote recruitment methods were used, all participants were provided with the same information consisting of a description of the study, the time commitment involved, contact information for research personnel, and a note that participating in this research study would in no way impact their position on the waitlist of the pain clinic. Recruitment continued until 100 participants at each site completed the survey and closed on October 31, 2022. The number of patients approached but not consenting to participate in the study was intended to be tracked; however, the pandemic impacted the tracking process. These data are not available at all sites except Ottawa (where the response rate was 55%). Written consent was collected from participants either in person on the day of their first appointment or by mail for those with remote appointments due to the COVID-19 protocols. Consent forms were stored securely according to the protocols defined by the local research ethics board.

Participants had various options to complete the survey in the clinic: (1) independently, in paper format (with large font); (2) independently, electronically (online) using a tablet that was provided in the clinic; or (3) with the assistance of the research assistant, who read the questions aloud and collected the answers. Furthermore, due to COVID-19 restrictions, some clinics only offered patients the option of completing the survey at home, either over the phone with the assistance of research staff or electronically using the link that was sent to them.

### Data Analysis

#### Quantitative Analysis

Descriptive analyses were performed using SPSS v28.0.1.1 (14). The results are presented as frequency counts and percentages. Gender-based breakdowns and comparisons across the sites were conducted using chi-square tests.

#### Qualitative Analysis

Data from an open-ended survey question, “Is there anything else you’d like to tell us?” were analyzed by two research team members (T.D. and R.H.) using a reflexive thematic analysis with axial coding.^29^ Initial coding was performed using a combination of descriptive, in vivo, and emotional coding; secondary coding was then performed to establish broader themes. T.D. and R.H. conducted this coding process independently and then met to discuss discrepancies, finalize themes, and, later, choose the quotes presented in this article. Findings and quotes chosen were discussed and validated in a meeting between T.D. and R.H. and adult patient partners with lived chronic pain experience.

## Results

### Quantitative

A total of 593 participants consented to take part in the survey. Not all questions were answered by everyone who consented. For questions with incomplete or partial responses, analyses were done on the data that were available. Response rates are reported for each question. Most participants completed the survey in English (*n* = 494, 83.3%) versus French (*n* = 99, 16.7%). Participant demographic characteristics are presented in Table 1. Our sample had a wide age range (9–92 years), with a median age of 59 years (range 17–92) in the total adult sample and 17 years (range 9–20) at the pediatric site. At each site, there were more female participants than male participants (63.4% vs. 36.3% in the adult total sample and 82.8% vs. 16.2% in the Montreal pediatric site). Most adult participants (*n* = 422, 86.8%) reported experiencing pain for over 1 year, with more than one-quarter (*n* = 128, 26.3%) reporting experiencing pain for over 10 years. For the pediatric population, the majority (*n* = 73, 73.8%) reported experiencing pain for between 6 months and 5 years. Among the adult sample, under a third reported being paid employees (*n* = 131, 27.6%) or retired (*n* = 144, 30.4%) as their main activity within the last week. Pediatric site participants largely reported going to school (*n* = 75, 82.4%). Most participants at all sites indicated having a regular health care provider (*n* = 455, 93.8% for the total adult sample and *n* = 85, 86.7% for the pediatric sample). At all sites, over 80% of participants reported having insurance coverage for prescription medication (*n* = 391, 80.5% for the total adult sample and *n* = 81, 83.5% for the pediatric sample).

**Table 1.** Patient demographic characteristics.

	Hamilton *(n* = 93)	London (*n* = 100)	Montreal pediatric clinic (*n* = 100)	Montreal (*n* = 101)	Ottawa (*n* = 99)	Winnipeg (*n* = 100)	Adult total (*n* = 493)
Gender	(*n* = 92)	(*n* = 99)	(*n* = 99)	(*n* = 99)	(*n* = 98)	(*n* = 99)	(*n* = 487)
Female	64 (69.6%)	62 (62.6%)	82 (82.8%)	64 (64.6%)	64 (65.3%)	55 (55.6%)	309 (63.4%)
Male	27 (29.3%)	37 (37.4%)	16 (16.2%)	35 (35.4%)	34 (34.7%)	44 (44.4%)	177 (36.3%)
Other	1 (1.1%)	0	0	0	0	0	1 (0.2%)
Prefer not to say	0	0	1 (1.0%)	0	0	0	0
Age	(*n* = 90)	(*n* = 98)	(*n* = 96)	(*n* = 99)	(*n* = 98)	(*n* = 99)	(*n* = 484)
Range	17–92	21–90	9–20	19–89	24–92	20–86	17–92
Median	59	59	17	56	55.5	62	59
Duration of pain	(*n* = 91)	(*n* = 98)	(*n* = 99)	(*n* = 99)	(*n* = 98)	(*n* = 100)	(*n* = 486)
Less than 3 months	0	2 (2%)	5 (5.1%)	3 (3%)	2 (2%)	1 (1%)	8 (1.6%)
3 months to 6 months	5 (5.5%)	11 (11.2%)	10 (10.1%)	2 (2%)	3 (3.1%)	2 (2%)	23 (4.7%)
6 months to 1 year	7 (7.7%)	7 (7.1%)	19 (19.2%)	9 (9.1%)	4 (4.1%)	6 (6%)	33 (6.8%)
1 year to 2 years	17 (18.7%)	23 (23.5%)	28 (28.3%)	22 (22.2%)	26 (26.5%)	11 (11%)	99 (20.4%)
3 years to 5 years	30 (33%)	19 (19.4%)	26 (26.3%)	22 (22.2%)	28 (28.6%)	22 (22%)	121 (24.9%)
6 years to 10 years	11 (12.1%)	16 (16.3%)	6 (6.1%)	12 (12.1%)	18 (18.4%)	17 (17%)	74 (15.2%)
More than 10 years	21 (23.1%)	20 (20.4%)	5 (5.1%)	29 (29.3%)	17 (17.3%)	41 (41%)	128 (26.3%)
Main activity last week	(*n* = 89)	(*n* = 95)	(*n* = 91)	(*n* = 96)	(*n* = 96)	(*n* = 98)	(*n* = 474)
Paid employment	29 (32.6%)	33 (34.7%)	1 (1.1%)	21 (21.9%)	27 (28.1%)	21 (21.4%)	131 (27.6%)
Vacation (from paid work)	1 (1.1%)	1 (1.1%)	0	5 (5.2%)	0	1 (1.0%)	8 (1.7%)
Looking for paid work	1 (1.1%)	0	2 (2.2%)	1 (1.0%)	2 (2.1%)	2 (2.0%)	6 (1.3%)
Going to school	2 (2.2%)	1 (1.1%)	75 (82.4%)	2 (2.1%)	1 (1.0%)	1 (1.0%)	7 (1.5%)
Caring for children	1 (1.1%)	1 (1.1%)	1 (1.1%)	3 (3.1%)	2 (2.1%)	1 (1.0%)	8 (1.7%)
Household work	5 (5.6%)	10 (10.5%)	2 (2.2%)	7 (7.3%)	2 (2.1%)	12 (12.2%)	36 (7.6%)
Retired	25 (28.1%)	31 (32.6%)	0	23 (24%)	31 (32.3%)	34 (34.7%)	144 (30.4%)
Long-term illness	9 (10.1%)	5 (5.3%)	0	22 (22.9%)	13 (13.5%)	12 (12.2%)	61 (12.9%)
Other	15 (16.9%)	12 (12.6%)	10 (11%)	12 (12.5%)	19 (19.8%)	14 (14.3%)	72 (15.2%)
Regular health care provider	(*n* = 92)	(*n* = 98)	(*n* = 98)	(*n* = 98)	(*n* = 97)	(*n* = 100)	(*n* = 485)
Yes	87 (94.6%)	93 (94.9%)	85 (86.7%)	86 (87.8%)	92 (94.8%)	97 (97%)	455 (93.8%)
No	5 (5.4%)	5 (5.1%)	13 (13.3%)	12 (12.2%)	5 (5.2%)	3 (3%)	30 (6.2%)
Insurance coverage for prescription medication	(*n* = 91)	(*n* = 99)	(*n* = 97)	(*n* = 99)	(*n* = 97)	(*n* = 100)	(*n* = 486)
Yes	75 (82.4%)	80 (80.8%)	81 (83.5%)	76 (77%)	81 (83.5%)	79 (79%)	391 (80.5%)
No	16 (17.6%)	19 (19.2%)	16 (16.5%)	23 (23%)	16 (16.5%)	21 (21%)	95 (19.5%)
Insurance coverage for long-term care^a^	(*n* = 87)	(*n* = 88)	(*n* = 87)	(*n* = 97)	(*n* = 96)	(*n* = 98)	(*n* = 466)
Yes	44 (50.6%)	37 (42%)	46 (52.9%)	32 (33%)	25 (26.0%)	31 (31.6%)	169 (36.3%)
No	43 (49.4%)	51 (58.0%)	41 (47.1%)	65 (67%)	71 (74.0%)	67 (68.4%)	297 (63.7%)

^a^Long-term care insurance can cover some of the costs of a care facility or a caregiver in a person’s own home following an accident or illness. In Canada, many long-term care facilities and home care services receive public funding; however, most also charge co-payments or extra fees for additional services that are not provided under the long-term plan.

Appointment characteristics are presented in Table 2. For nearly all participants (*n* = 546, 93.2%), chronic pain of more than 3 months’ duration was the main reason for visiting the chronic pain clinic. In the adult sample, most participants were referred to the pain clinic by their usual primary care provider (PCP; *n* = 261, 53.6%) or a specialist physician (*n* = 181, 37.2%). At the Montreal pediatric site, a larger proportion of participants (*n* = 66, 66.7%) were referred by a specialist physician as opposed to their usual PCP (*n* = 16, 16.2%).

**Table 2.** Appointment characteristics.

	Hamilton (*n* = 93)	London (*n* = 100)	Montreal pediatric clinic (*n* = 100)	Montreal (*n* = 101)	Ottawa (*n* = 99)	Winnipeg (*n* = 100)	Adult total (*n* = 493)
Referrer	(*n* = 92)	(*n* = 98)	(*n* = 99)	(*n* = 99)	(*n* = 98)	(*n* = 100)	(*n* = 487)
Patient’s usual primary care provider	43 (46.7%)	64 (65.3%)	16 (16.2%)	38 (38.4%)	53 (54.1%)	63 (63%)	261 (53.6%)
Patient’s usual nurse practitioner	0	1 (1.0%)	1 (1.0%)	1 (1.0%)	1 (1.0%)	1 (1.0%)	4 (0.8%)
Walk-in clinic doctor	1 (1.1%)	1 (1.0%)	1 (1.0%)	1 (1.0%)	0	2 (2.0%)	5 (1.0%)
Emergency room doctor	1 (1.1%)	2 (2.0%)	9 (9.1%)	4 (4.0%)	3 (3.1%)	1 (1.0%)	11 (2.3%)
Specialist physician	40 (43.5%)	27 (27.6%)	66 (66.7%)	50 (50.5%)	39 (39.8%)	24 (24.0%)	181 (37.2%)
“I don’t know”	0	0	2 (2.0%)	0	0	6 (6%)	6 (1.2%)
Other	7 (7.6%)	3 (3.1%)	4 (4.0%)	5 (5.1%)	2 (2.0%)	2 (2.0%)	19 (3.9%)
Main reason(s) for visit	(*n* = 92)	(*n* = 98)	(*n* = 99)	(*n* = 99)	(*n* = 98)	(*n* = 100)	(*n* = 487)
New pain (acute, less than 3 months)	4 (4.3%)	7 (7.1%)	6 (6.1%)	6 (6.1%)	2 (2.0%)	4 (4.0%)	23 (4.7%)
Chronic pain (more than 3 months)	85 (92.4%)	87 (88.8%)	93 (93.9%)	92 (92.9%)	91 (92.9%)	98 (98%)	453 (93.0%)
Cancer pain	2 (2.2%)	0	0	1 (1.0%)	1 (1.0%)	4 (4.0%)	8 (1.6%)
Postoperative pain	8 (8.7%)	5 (5.1%)	2 (2.0%)	10 (10.1%)	20 (20.4%)	5 (5.0%)	48 (9.9%)
Other	10 (10.9%)	3 (3.1%)	1 (1.0%)	7 (7.1%)	7 (7.1%)	4 (4.0%)	31 (6.4%)
Method of transport to appointment	(*n* = 92)	(*n* = 99)	(*n* = 94)	(*n* = 97)	(*n* = 94)	(*n* = 99)	(*n* = 481)
Regular bus	2 (2.2%)	1 (1.0%)	6 (6.4%)	10 (10.3%)	1 (1.1%)	4 (4.0%)	18 (3.7%)
Special transport (i.e., handicap-accessible bus)	2 (2.2%)	3 (3.0%)	1 (1.1%)	7 (7.2%)	0	5 (5.1%)	17 (3.5%)
Someone came with me or dropped me off	28 (30.4%)	51 (51.5%)	26 (27.7%)	37 (38.1%)	6 (6.4%)	41 (41.4%)	163 (33.9%)
Walked	0	0	0	1 (1.0%)	1 (1.1%)	0	2 (0.4%)
By personal car	52 (56.5%)	42 (42.4%)	43 (45.7%)	29 (29.9%)	18 (19.1%)	44 (44.4%)	185 (38.5%)
Taxi/drive sharing service	2 (2.2%)	2 (2.0%)	0	5 (5.2%)	2 (2.1%)	2 (2.0%)	13 (2.7%)
Other	6 (6.5%)	1 (1.0%)	18 (19.1%)	8 (8.2%)	66 (70.2%)	3 (3.0%)	84 (17.5%)
Reasons for missing a health care appointment in the past	(*n* = 92)	(*n* = 99)	(*n* = 98)	(*n* = 99)	(*n* = 98)	(*n* = 100)	(*n* = 488)
Didn’t have a way to get to the appointment (no transportation)	5 (5.4%)	4 (4.0%)	2 (2.0%)	13 (13.1%)	6 (6.1%)	8 (8.0%)	36 (7.4%)
It was too far away	2 (2.2%)	2 (2.0%)	5 (5.1%)	7 (7.1%)	5 (5.1%)	3 (3.0%)	19 (3.9%)
It was too expensive to get there	0 (0%)	3 (3.0%)	0 (0%)	5 (5.1%)	3 (3.1%)	3 (3.0%)	14 (2.9%)
I had no one to take me that day	7 (7.6%)	4 (4.0%)	1 (1.0%)	10 (10.1%)	5 (5.1%)	3 (3.0%)	29 (5.9%)
My pain was too severe	12 (13.0%)	10 (10.1%)	9 (9.2%)	22 (22.2%)	18 (18.4%)	18 (18.0%)	80 (16.4%)
I had to care for another person	2 (2.2%)	3 (3.0%)	0 (0%)	2 (2.0%)	3 (3.1%)	4 (4.0%)	14 (2.9%)
I couldn’t pay for the visit (e.g., no provincial insurance)	3 (3.3%)	0 (0%)	1 (1.0%)	6 (6.1%)	3 (3.1%)	2 (2.0%)	14 (2.9%)
My communication needs could not be met (e.g., interpreter)	0 (0%)	1 (1.0%)	0 (0%)	1 (1%)	0 (0%)	0 (0%)	2 (0.4%)
The office could not accommodate my physical needs (e.g., wheelchair, lifts)	0 (0%)	0 (0%)	0 (0%)	3 (3.0%)	1 (1.0%)	2 (2.0%)	6 (1.2%)
Forgot about the appointment	9 (9.8%)	11 (11.1%)	2 (2.0%)	6 (6.1%)	8 (8.2%)	6 (6.0%)	40 (8.2%)
I couldn’t attend because of work or school/exams	8 (8.7%)	7 (7.1%)	7 (7.1%)	4 (4.0%)	6 (6.1%)	3 (3.0%)	28 (5.7%)
Other	9 (9.8%)	4 (4.0%)	4 (4.1%)	5 (5.1%)	6 (6.1%)	8 (8.0%)	32 (6.6%)
Immediate support (family or friends) who help with health care appointments if needed	(*n* = 92)	(*n* = 97)	(*n* = 98)	(*n* = 100)	(*n* = 97)	(*n* = 99)	(*n* = 485)
Yes	82 (89.1%)	92 (94.8%)	93 (94.9%)	78 (78.0%)	85 (87.6%)	88 (88.9%)	425 (87.6%)
No	10 (10.9%)	6 (6.2%)	2 (2.0%)	16 (16.2%)	8 (8.2%)	7 (7.1%)	47 (9.7%)
Other	0	1 (1.0%)	3 (3.1%)	5 (5.0%)	4 (4.1%)	4 (4.0%)	14 (2.9%)

**Figure 1.**
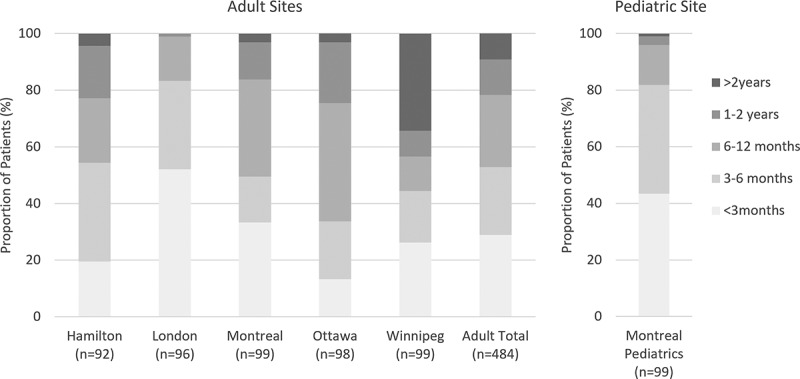
Chronic pain clinic wait times across the six participating sites.

Figure 1 presents wait times for chronic pain clinic appointments across the six participating sites, along with the total adult sample. Across all adult sites, just over half of participants (*n* = 256, 52.8%) reported waiting less than 6 months, one-quarter (*n* = 123, 25.4%) reported waiting between 6 months to 1 year, and one-fifth (*n* = 106, 21.9%) reported waiting over 1 year. The highest proportion of patients reporting waiting less than 6 months for a chronic pain appointment was in London (*n* = 80, 83.3%) and the Montreal pediatric clinic (*n* = 81, 81.8%). The highest proportion of patients reporting waiting over a year was in Winnipeg (43.4%), with over a third (*n* = 34, 34.3%) waiting over 2 years. No gender differences were observed in how long participants waited for their first appointment at the pain clinics (*P* > 0.05).

**Figure 2.**
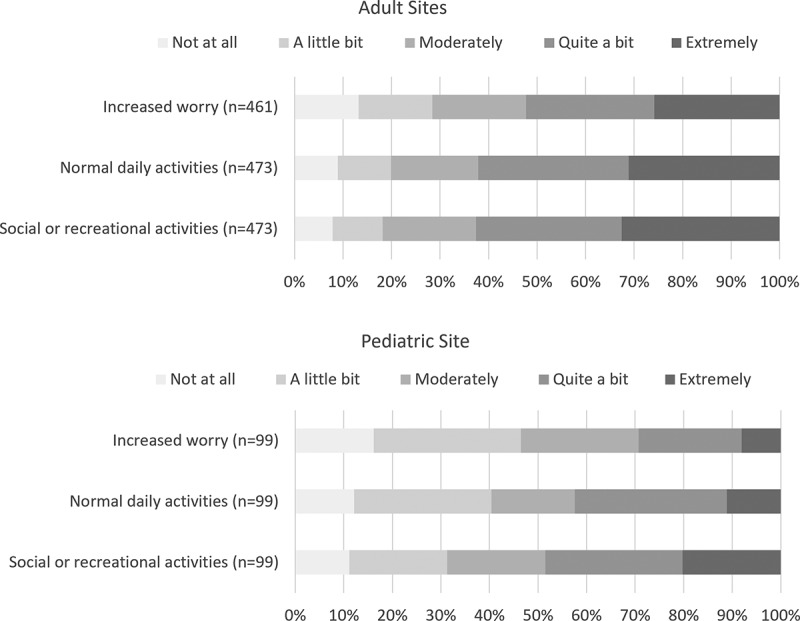
Impact of chronic pain on quality of life measures.

The impact of chronic pain on quality of life measures is illustrated in Figure 2. Across all sites, between 52% to 63% of adults and between 30% to 49% of pediatric respondents reported that their chronic pain increased worry and limited their ability to carry out daily activities and participate in usual social activities “quite a bit” or “extremely.”

**Figure 3.**
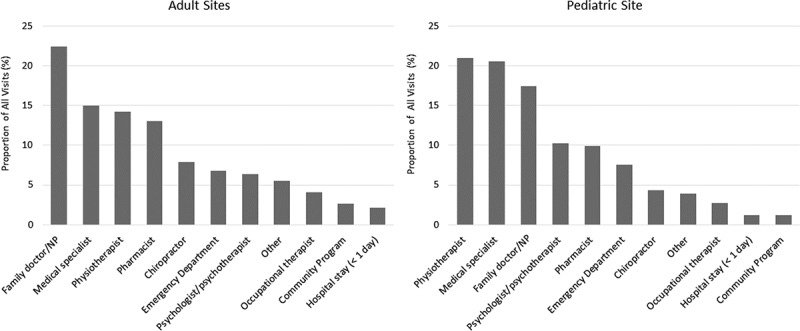
Other health care professionals seen by patients while waiting for their chronic pain clinic appointment. NP = nurse practitioner.

While waiting for their appointment at the pain clinics, participants reported visiting other health care providers and settings for pain care (Figure 3). The most visited health care professionals for all adult sites and the pediatric site included family doctor/nurse practitioner (69.3%, *n* = 407), medical specialists (50.3%, *n* = 295), and physiotherapists (48.3%, *n* = 283).

### Qualitative

The burden of chronic pain was further expressed through the open text responses to the open-ended question, “Is there anything else you want to tell us?” Two hundred and thirty-one of 593 participants left a response (39.0%). One hundred and seventy-seven responses noted the impact of participants’ chronic pain on their physical and mental health and well-being, attempts to access treatments for their pain, and specific barriers they faced in their attempts to access specialist advice (76.6%). We grouped these into eight distinct themes: system navigation issues, administrative issues, decreased quality of life, distress, self-advocacy, coping strategies, communication, and distrust. A brief description of each theme with representational quotes is presented in Table 3.

**Table 3.** Themes from qualitative analysis of free text responses.

Theme	Description	Sample quote
System navigation issues	Going to and/or bouncing between multiple clinics, physicians, specialties to try to establish a diagnosis and/or manage pain. Trying to figure out how to get help with or without physician support; unclear path to pain clinics	“Attempting to access different specialists, even with the help of my GP has been a slow process where my condition continued to degrade and where considerable expenses have been incurred and significant impact of my life.”
Administrative issues	Problems with referrals (e.g., lost referrals or lengthy and/or complicated process)	“As time went on I called the office whereby I was informed that I was not on the referral list. After contacting the hospital it was determined that the referral was not done and this error was corrected.”
Decreased quality of life	Negative personal impacts of living with chronic pain on lifestyle and activities of daily living	“I have been drinking more alcohol, and taking Diloted (carefully) when needed to manage the pain. It is so horrible. I have left my life. I have 2 kids. … […] My 8 yr old only know me as a person who cannot do [any]thing because of pain.”
Distress	A sense of feeling alone and unsupported, and a loss of hope	“I can’t sleep, I can’t work, I’m in so much pain. I can’t get medication that I need. I feel devalued as a human and I feel like a junkie.”
Self-advocacy	Patients advocating for themselves by initiating the pain clinic referral via their request and/or contacts pain clinic for information after referral for scheduling	“I had to call repeatedly to ensure that progress was being made. I finally received an appointment for June 2022. Had I not repeatedly followed up I would still be waiting while dealing with unmanaged chronic pain.”
Coping strategies	Range of techniques and efforts patients use to manage their pain	“Prior to being referred to the pain clinic I had also invested in chiropractic treatment, an inversion table, tens type device, ice packs and heating pads, topical ointments, etc., in an attempt to control the pain.”
Communication	Patients not knowing what to expect, feeling uninformed about the true length of wait times, whether referrals were made, the types of issues they can bring to the pain clinic, and what the pain clinic can offer	“I called many times over the summer and fall to get different answers. I wasn’t told the real truth of the wait time until the fall. I was then told it would still be 18 months probably! This is disgusting. Specialists and people who are answering the calls should be honest!! Stop giving people in pain HOPE, when that HOPE will not be coming for years!”
Distrust	Patients’ perception of not being believed or their concerns not being taken seriously by one or more physicians (paternalistic model of doctor-patient interactions)	“As I was told many times it’s impossible that I’m in this much pain, well I live it everyday and the pain is real.”

Patients described the consequences associated with waiting to access specialist care due to a health system that is difficult to navigate, even with the help of their family doctor. Above all, participants voiced the impact their chronic pain has had on their quality of life and the frustrations with wait times for pain clinic appointments. Some also highlighted the financial burden of seeking alternative treatments to cope with their pain while waiting for an appointment at a pain clinic. Others expressed a sense of hopelessness and feeling alone and unsupported. Participants also expressed their perceptions of being stigmatized and not being believed with respect to the extent of their chronic pain and not being taken seriously by one or more physicians.

## Discussion

Our study found that patients continue facing barriers to timely access to specialty care for their pain, similar to what we found 9 years ago through a survey of patients with chronic pain at a single site in Ontario.^21^ Just over half of adult survey respondents in the present study reported waiting 6 months or less for their first appointment at the pain clinic, with one in five reporting waiting over 1 year. During the waiting period, around 80% of adult and 60% of pediatric patients reported having experienced some impact of chronic pain on their quality of life due to increased worry and limitations to normal daily activities and social or recreational activities. A fuller picture of the personal impacts of waiting while living with chronic pain emerged based on responses to an open-ended question about patient experiences, where they expressed deep frustration with wait times for pain clinic appointments and the difficulties associated with waiting to access specialist care.

### Wait Times, Benchmarks, and Patient Preferences

Our findings regarding current wait times are consistent with the results from other Canadian studies, which showed that the median wait time for a first appointment in public multidisciplinary pain clinics remained at around 6 months over the past 18 years.^22,23^ This means that while 50% of patients are seen in 6 months or less, the other 50% wait much longer, in some cases 2 years or more, to gain access to specialized treatment for their pain. This delayed treatment of chronic pain in Canada surpasses recommended benchmarks by guidelines for chronic pain management,^30,31^ which are based on evidence showing deterioration in health-related quality of life, increased pain, and increasing depression in patients waiting more than 6 months from the time of referral.^19^ Some studies demonstrated this deterioration as early as 5 weeks into the waiting period.^19^

In terms of patient preferences, our earlier study showed that for 83% of patients with chronic pain waiting for their first appointment at a pain clinic, the ideal wait time was less than 3 months.^21^ In that same study, only a third of patients received care within 3 months of being referred, which is similar to the current study, where only 29% of adult participants and 43% of pediatric participants reported receiving an appointment within 3 months. It is encouraging that 80% of pediatric patients in our sample were seen within the guideline-directed time of 6 months or less since their referral, given the negative impacts of chronic pain on a child’s physical health and adverse long-term outcomes during the transition to adulthood.^32–34^

We observed substantial variability in wait times between the adult sites. Though not directly assessed in this study, these differences may be related to the variability in the pain clinics’ ability to meet clinical demands. It is generally known that not all referrals to the pain clinic are accepted. Though we do not know the referral rejection rates of the clinics that participated in the study, it is possible that some clinics may only appear to be performing better in terms of wait times because their referral rejection rates are higher, potentially making the volume of referrals more manageable. Past research also points to the importance of administrative processes and availability of resources (in addition to patient volumes) as factors contributing to the variance in wait times.^35^

### Impact on Quality of Life

In terms of quality of life, most survey respondents in our study reported being negatively affected by their chronic pain while waiting for access to specialized services. The qualitative analysis of free-text comments revealed the complex and multifaceted nature of the experiences of those living with chronic pain while awaiting treatment. Patients described their pain not only as a physical burden but also as a psychological burden that affected many aspects of their lives. They also expressed feeling isolated, misunderstood, and not believed by their family, friends, and health care providers, which often led to a sense of frustration and hopelessness. These findings are consistent with previous research on the complex interactions between biological, psychological, and social factors of the chronic pain experience^36^ and the tremendous toll of pain on social functioning.^37–39^

### Reliance on Other Health Providers

Given the long wait times for pain specialists, many patients with chronic pain turn to other health care providers in attempts to manage their pain. The most visited health care professionals for the adult participants were family doctors and/or nurse practitioners, and for the pediatric participants the most visited health care professionals were physiotherapists. Other frequently visited providers included allied health services, such as pharmacists, chiropractors, psychologists, and psychotherapists. The value of allied health services and offering multidisciplinary management for chronic pain has been recognized in the literature,^40,41^ along with the importance of self-management approaches in chronic pain management.^42^ In the free-text responses, our participants highlighted a range of strategies they used to manage their pain. These included physical activity, relaxation techniques, mindfulness-based interventions, and medications, all of which are supported by prior research.^42^ Some participants noted financial burdens from seeking alternative treatments to cope with their chronic pain.

### Implications

Our findings highlight the detrimental impacts of long wait times to obtain chronic pain specialty care experienced by over half of patients living with chronic pain in our sample. These patients expressed frustration with current wait times and not understanding the reasons for their pain or why they need to wait so long. Transparency regarding the realistic duration of wait times to see pain specialists seems to be lacking, yielding disillusioned patients who lose trust in the medical system.

Our study points to the urgent need to better support patients during the waiting period. In this regard, expanding technologies such as eConsult hold great promise. eConsult is a secure online application that allows PCPs to communicate electronically with specialists from a variety of specialty groups. By facilitating a prompt communication between PCPs and specialists, eConsult can help mitigate the negative effects of long wait times and dramatically improve the patient experience for those living with chronic pain who have access to primary care.^43,44^ Furthermore, the reliance on other health providers and coping strategies reported by our respondents suggests the need for increased resources for allied health providers, including rehabilitation services (e.g., physiotherapy, occupational therapy) and mental health services (e.g., psychologists, social workers). In addition, dissemination of self-management approaches in chronic pain management, such as the Power Over Pain Portal,^45^ is important and could be helpful. The Power Over Pain Portal is a new online tool to support patients living with chronic pain and was created by a pan-Canadian collaboration of people living with pain, clinicians, researchers, and representatives of community organizations that offer free, evidence-based online resources for the management of pain, as well as mental health and substance use health resources.

### Strengths and Limitations

Our study and survey instrument were co-designed with patient partners, clinicians, and researchers, who also participated in data analysis and interpretation.^27^ Such a level of integration of the perspectives of people with lived experience throughout the entire research process offers important benefits. These include assurance that the survey was responsive to the needs of people living with chronic pain and sensitive to local community contexts, that the outcomes captured were meaningful to the participating communities, and that the findings can provide a solid basis for informing program and policy development.

Our study also has some limitations. The survey took place in six pain clinics in Canada, which is a relatively small sample given that there are at least 97 publicly funded multidisciplinary pain treatment facilities across Canada.^23^ Different clinics offer different services and have different triage processes and referral practices, all of which are expected to impact wait times and patient experience. These processes were affected by the COVID-19 pandemic, which caused disruptions to services and clinics across the country and may have contributed to the observed wait times, the quality of life of patients with chronic pain, and the observed reliance on other health care providers.^46,47^ The participating clinics were publicly funded (except for the Montreal pediatric site, which is funded through private donors, such as the Louise and Alan Edwards Foundation); hence, our findings may not be transferrable to other contexts (e.g., private, third-party funded). Our study sample included more females than males, and this difference was especially pronounced within the pediatric sample. Though this is in line with past research^7^ and similar female-to-male ratios have been reported in adults^48^ and children,^49^ this gender bias may also reflect sex-based disparities in research participation, access to health care, and resource utilization among persons living with chronic pain. To limit the number of survey questions and respondent burden, we only asked the participants about their gender, not biological sex. Though both biological sex and gender are important,^50^ it is difficult to dissociate the biological, psychological, and social differences between men and women with respect to pain, because these differences are interrelated.^51^ Emerging evidence shows that gender identity may play a more significant role in pain sensation than biological sex.^52^ Furthermore, gendered norms about men and women with chronic pain and the unintended gender bias in the treatment of pain not embedded in biological differences but gendered norms have been described.^51^ All in all, these considerations underline the importance of including both sex- and gender-inclusive analyses to advancing pain research. We included incomplete or partial survey responses to maximize all respondents’ contributions. Though this may limit generalizability of the findings, it may reflect the challenges of answering questionnaires with multiple questions and multiple-choice answers for people living with chronic pain. We also observed a low response rate to an open-ended question that was subjected to a qualitative analysis. Though completing responses to open-ended questions requires more time and mental effort and thus may have been more taxing on those living with chronic pain, it may indicate nonresponse bias. Given our intent to minimize the number of questions, certain demographic information was not collected. For example, there were no questions pertaining to racialization,^13^ indigeneity,^12^ or socioeconomic position,^10^ all of which have been shown to be associated with increased prevalence of chronic pain as well as with greater barriers in accessing care. Future survey-based research on chronic pain populations and access to pain clinics should directly address these equity-seeking populations. Our survey instrument, though co-designed with patient partners, researchers, and health care providers, which adds to its face validity, was not validated in the population of people living with chronic pain. As such, it is possible that the survey questions did not accurately measure the intended constructs. Finally, it should be noted that a vast majority of participants in our study reported having a regular health care provider, which is not the case for about one in five Canadians, according to a recent national survey.^53^ Unfortunately, this patient population may not benefit from the eConsult service owing to limited access to a PCP.

## Conclusion

Chronic pain is a pervasive issue that affects millions of people worldwide. Access to specialized pain clinics is recommended to improve management of chronic pain. This study provides real-time regional snapshots into wait times and access issues experienced by patients with chronic pain and highlights the detrimental impact of waiting while living with chronic pain on quality of life. These results will be crucial in understanding what modifications to existing services are needed and to inform the creation of tailored chronic pain services, such as eConsult, in each region.

## Supplementary Material

Supplemental Material

## Data Availability

The data that support the findings of this study are available from the corresponding author upon reasonable request.
