# Supplementary material for: Patient-Reported Wait Times and the Impact of Living with Chronic Pain on their Quality of Life: A Waiting Room Survey in Chronic Pain Clinics in Ontario, Manitoba, and Quebec
Source: Can J Pain. 2024 Apr 22;8(1):2345612. doi: 10.1080/24740527.2024.2345612 (PMC11185187; doi:10.1080/24740527.2024.2345612)
Supplement: Supplemental Material [file UCJP_A_2345612_SM3047.docx]

Waiting Room Survey for Chronic Pain


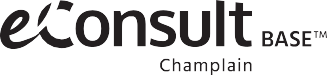


**CONFIDENTIAL**

# What is/are the main reason(s) you are here to see the specialist?

- - New pain

(acute pain, less than 3 months)

- - Chronic pain (more than 3 months)
  - Cancer pain
  - Post-operative pain
  - Other (please specify):

# How long have you been experiencing this pain?

- - Less than 3 months
  - 3 months to 6 months
  - 6 months to 1 year
  - 1 year to 2 years
  - 3 years to 5 years
  - 6 years to 10 years
  - More than 10 years

# Who referred you to this pain clinic?

- - My usual family doctor
  - My nurse practitioner
  - A walk-in clinic doctor
  - An emergency room doctor
  - Another specialist doctor
- I don’t know
- Other (please specify):

# Do you have a regular health care provider? By this, we mean one health professional that you regularly see or talk to when you need care or advice for your health?

- - No — please go to question 6 □ Yes — continue to question 5

# Is that regular health care provider a . . . ?

- - Family doctor or general practitioner
  - Medical specialist such as a cardiologist or a pediatrician
  - Nurse practitioner
- Other (please specify):

# How long have you been waiting for this appointment since you were referred to this pain clinic?

- - Less than 3 months
  - 3 months to 6 months
  - 6 months to 1 year
  - 1 year to 2 years
  - 2 years or more

# While waiting for this appointment at the pain clinic, has your pain . . .

Not at all A little bit Moderately Quite a bit Extremely

Caused you to miss work/school

□ □ □ □ □

Caused you to worry more □ □ □ □ □

Limited your ability to carry out normal daily activities (e.g. preparing/eating meals, household tasks, sleeping, personal hygiene, etc.)

□ □ □ □ □

Limited your ability to participate in your usual social or

recreational activities

□ □ □ □ □

| Community program (e.g., self- management/educational support) | □ | □ | □ | □ |
| --- | --- | --- | --- | --- |
| Other (specify): | □ | □ | □ | □ |

| **8. While you were waiting for this appointment, what other**  **health care professionals/settings have you seen for your pain?**  0 times 1–2 times | 3–5 times | 5+ times |
| --- | --- | --- |
| Your family doctor/nurse practitioner □ □ | □ | □ |
| Emergency Department □ □ | □ | □ |
| Hospital stay (more than 1 day) □ □ | □ | □ |
| Medical specialist □ □ | □ | □ |
| Physiotherapist □ □ | □ | □ |
| Occupational therapist □ □ | □ | □ |
| Psychologist/psychotherapist □ □ | □ | □ |
| Chiropractor □ □ | □ | □ |
| Pharmacist □ □ | □ | □ |

# Is there anything else that you would like to tell us?

1. **How did you arrive to the pain clinic today?**
   - Regular bus
   - Special transport (i.e. handicap bus)
   - Someone came with me/dropped me off
   - Walked
   - Biked

- Personal car
- Taxi/drive sharing service
- Other (please specify):

# Have you ever missed a health care appointment due to any of the following? Check all that apply. If none apply, please go to question 12.

- - Didn’t have a way to get to the appointment (no transportation)
  - It was too far away
  - It was too expensive to get there
  - I had no one to take me that day
  - My pain was too severe
  - I had to care for another person
  - I couldn’t pay for the visit (e.g. no provincial insurance)
  - My communication needs could not be met (e.g. interpreter)
  - The office could not accommodate my physical needs (e.g. wheelchair, lifts)
  - Forgot about the appointment
  - I couldn’t attend because of work or school/exams
  - Other (please specify):

# Do you have immediate support, such as family or friends, who help you with your health care appointments if needed?

- - Yes
  - No
  - Other (please specify):

# In what year were you born? (Enter 4-digit birth year; for example, 1976)

|  |  |  |  |
| --- | --- | --- | --- |

1. **What is your gender?**
   - Female
   - Male

- Other
- Prefer not to answer

# Please provide the first 3 digits of your postal code

|  |  |  |
| --- | --- | --- |

1. **Last week, was your main activity working at a paid job or business, looking for paid work, going to school, caring for children, household work, retired or something else?**
   - Working at a paid job or business
   - Vacation (from paid work)
   - Looking for paid work
   - Going to school (including home schooling and vacation from school)
   - Caring for children
   - Household work
   - Retired
   - Maternity/paternity leave
   - Long term illness
   - Volunteering
   - Care-giving other than for children
   - Other (please specify):

# Do you have insurance that covers all or part of the cost of your prescription medications?

- - Yes □ No

# If yes, is it...?

- - A government-sponsored plan
  - An employer-sponsored benefit plan
  - A plan sponsored through an association such as a union, trade association or student organization
- Other, such as your own private plan purchased from an insurance company

# Do you have insurance that covers all or part of your long-term care costs, including home care?

- - Yes □ No

# If yes, is it...?

- - A government-sponsored plan
  - An employer-sponsored benefit plan
  - A plan sponsored through an association such as a union, trade association or student organization
- Other, such as your own private plan purchased from an insurance company

# Can we contact you about participating in the future research studies?

- - Yes □ No

**If yes, please provide your first and last name, telephone number, and email address below First name:**

|  |  |  |  |  |  |  |  |  |  |  |  |  |  |  |  |  |  |  |  |  |
| --- | --- | --- | --- | --- | --- | --- | --- | --- | --- | --- | --- | --- | --- | --- | --- | --- | --- | --- | --- | --- |

**Last name:**

|  |  |  |  |  |  |  |  |  |  |  |  |  |  |  |  |  |  |  |  |  |
| --- | --- | --- | --- | --- | --- | --- | --- | --- | --- | --- | --- | --- | --- | --- | --- | --- | --- | --- | --- | --- |

**Telephone number:**

( ) -

|  |  |  |
| --- | --- | --- |

|  |  |  |
| --- | --- | --- |

|  |  |  |  |
| --- | --- | --- | --- |

# When is the best day/time to reach you over the phone?

**Email address:**

|  |  |  |  |  |  |  |  |  |  |  |  |  |  |  |  |  |  |  |  |  |
| --- | --- | --- | --- | --- | --- | --- | --- | --- | --- | --- | --- | --- | --- | --- | --- | --- | --- | --- | --- | --- |

**Note:** In order to maintain confidentiality and anonymity, this page will be separated from the rest of the pages of this questionnaire and stored in a separate file.
